# Aristolochic Acid Causes Albuminuria by Promoting Mitochondrial DNA Damage and Dysfunction in Podocyte

**DOI:** 10.1371/journal.pone.0083408

**Published:** 2013-12-13

**Authors:** Yang Zhou, Xueqin Bian, Li Fang, Weichun He, Chunsun Dai, Junwei Yang

**Affiliations:** Center for Kidney Disease, 2^nd^ Affiliated Hospital, Nanjing Medical University, Nanjing, Jiangsu Province, China; University of Houston, United States of America

## Abstract

Aristolochic acid nephropathy, initially found in patients intaking of slimming herbs containing aristolochic acid (AA), was previously considered as a progressive renal interstitial fibrosis and urothelial malignancy. However, the presence of albuminuria in some patients with AAN suggests that AA may also damage the glomerular filtration barrier. In this study, mice AAN model was generated by daily administration of aristolochic acid I sodium salt intraperitoneally at a dose of 6 mg/kg body weight for 3 days. All of the mice developed heavy albuminuria at day 3 and 7 after receiving AA. In the mice received AA, morphologic change of glomeruli was minor under light microscopy but podocyte foot-process effacement was evident under electron microscopy. In mitochondria isolated from kidney, prominent mitochondrial DNA (mtDNA) damage was accompanied with marked decrease of mtDNA copy number and mitochondrial protein expression level. Similar to those in vivo results, AA treatment impaired the filtration barrier function of cultured podocytes. AA promoted mtDNA damage, decreased mtDNA copy number and mitochondrial protein expression in cultured podocytes. In addition, AA treatment also decreased ATP content, oxygen consumption rate and mitochondrial membrane potential as well as increased cellular reactive oxygen species in cultured podocytes. This study highlighted that AA could induce podocyte damage and albuminuria, which may be mediated by promoting mtDNA damage and mitochondrial dysfunction in podocytes.

## Introduction

More than two decades ago, a clinical manifestation of severe interstitial nephritis that rapidly progressed to end-stage renal disease was found in patients taking weight-reducing pills [1]. Very soon, an epidemiological survey identified an ingredient in those pills called aristolochic acid [2] which resulted in rapid progressive interstitial renal fibrosis [3,4]. Since then, AA-induced renal disease was designated as aristolochic acid nephropathy (AAN) [1,3–6]. Despite the recognition of the etiology of AAN, the development of AAN seemed hard to prevent and the incidence of AAN was still probably underestimated, as numerous widely used traditional medicines contain AA [7,8]. AA is also easily available [9]. In addition, AA could be intake from environment which resulted in a slow progressive interstitial disease and an increased frequency of urothelial cancer [10,11]. These reasons highlight the importance and necessity of investigating the mechanism and treatment of AAN.

During these years, AAN was pathophysiologically considered as a progressive renal interstitial fibrosis and urothelial malignancy [12–15]. However, the presence of albuminuria in some patients with AAN and animal received AA [12,13,15,16] suggests that AA may also damage the glomerular filtration barrier. Here, mice administered with AA developed heavy albuminuria. Podocyte foot-process effacement was evident under electron microscopy. Similar to those in vivo results, AA treatment could impair the filtration barrier function of cultured podocytes. AA induced cell damage and carcinogenesis are mainly attributed to the forming of DNA adducts with metabolized AA in patients [17]. In this study, in mitochondria isolated from cultured podocytes or kidney, prominent mitochondrial DNA (mtDNA) damage was observed after AA, accompanied with marked decrease of mtDNA copy number and mitochondrial protein expression level. In cultured podocytes, AA treatment could impair mitochondrial functions as demonstrated by decreasing ATP content, oxygen consumption rate and mitochondrial membrane potential as well as increasing cellular reactive oxygen species.

## Results

### AA administration leads to albuminuria in mice

**Figure 1:**
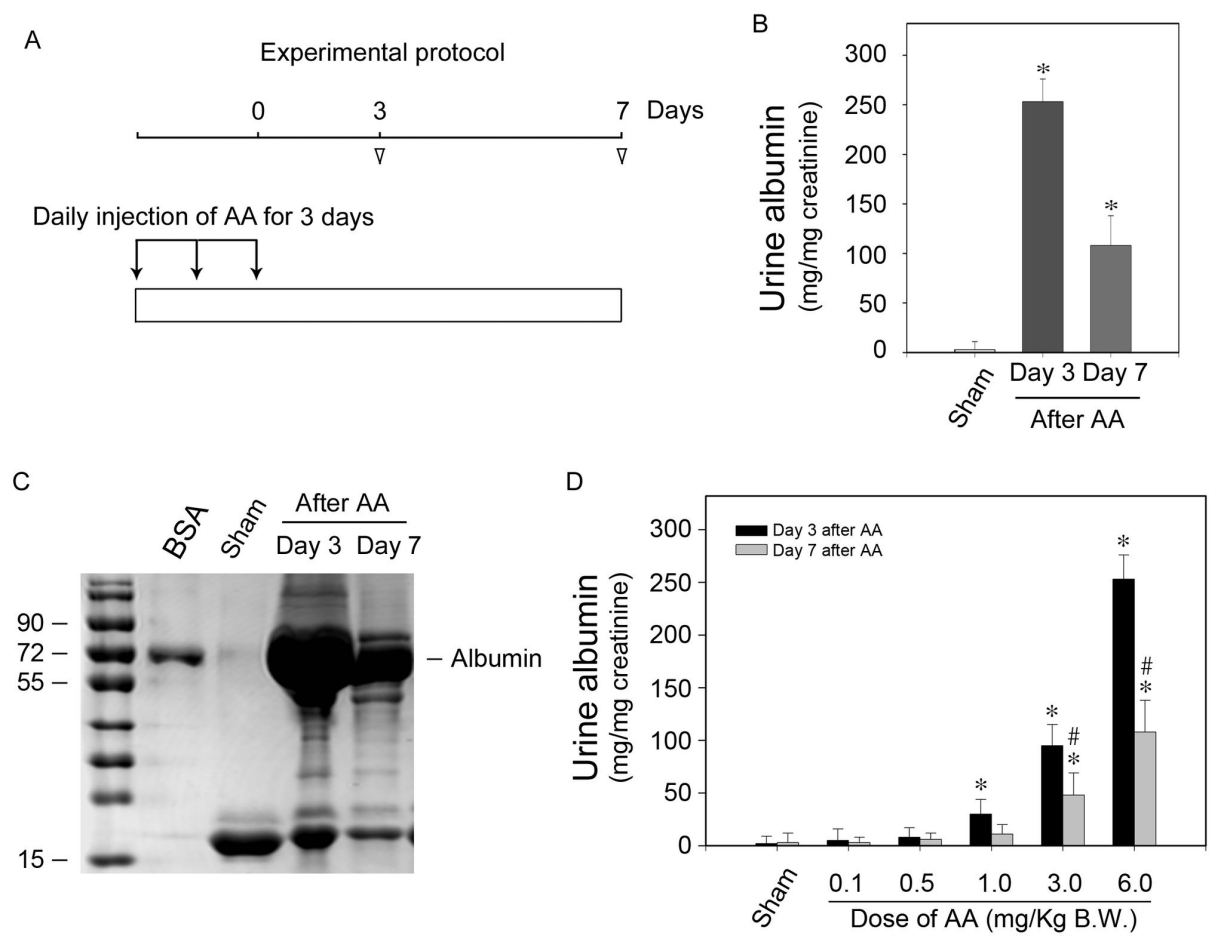
AA administration leads to albuminuria in mice. (A) Schematic illustration of experimental protocols. Arrows indicate the timing of AA injections. Arrowheads depict the time points when mice were killed after AA administration. (B) Urinary albumin levels at different time points after 6mg/kg body weight of AA administration. Urinary albumin data after correction to creatinine are presented as mean ± SEM. * P < 0.05 versus sham (n = 7). (C) SDS-PAGE demonstrates urine proteins at different time points after AA administration. Urine samples (pooled from 7 mice) were separated on 10% SDS-PAGE after corrected to creatinine. BSA (1µg) was loaded as a positive control. (D) AA was administered at different doses as indicated. Urinary albumin levels at different time points after different doses of AA administration. Urinary albumin data after correction to creatinine are presented as mean ± SEM. * P < 0.05 versus sham, # P < 0.05 verse day 3 after AA (n = 7).

Figure 1A illustrates the experimental protocol. AA was daily administered intraperitoneally at a dose of 6mg/kg body weight for three days. Simultaneously, sham mice were injected with same volume of saline with its pH value adjusted by hydrochloric acid. Urine samples were collected and albumin/creatinine ratio was examined. As shown in Figure 1B, all of the mice developed albuminuria at both day 3 and day 7 after receiving AA. Consistently, separation of the urine samples by SDS-PAGE after normalizing their creatinine levels revealed that albumin was the predominant constituent of urine protein after receiving AA (Figure 1C). To determine the specificity of AA on inducing albuminuria, AA was administered at increasing doses from 0.1 to 6mg/kg body weight. As shown in Figure 1D, urine excretion level of albumin was increased at an AA dose-dependent manner and as low as 1mg/kg of AA leaded to markedly increased albuminuria at day 3. These results suggested that AA could dose-dependently induce albuminuria in mice received AA.

### AA causes podocyte foot-process effacement and detachment

**Figure 2:**
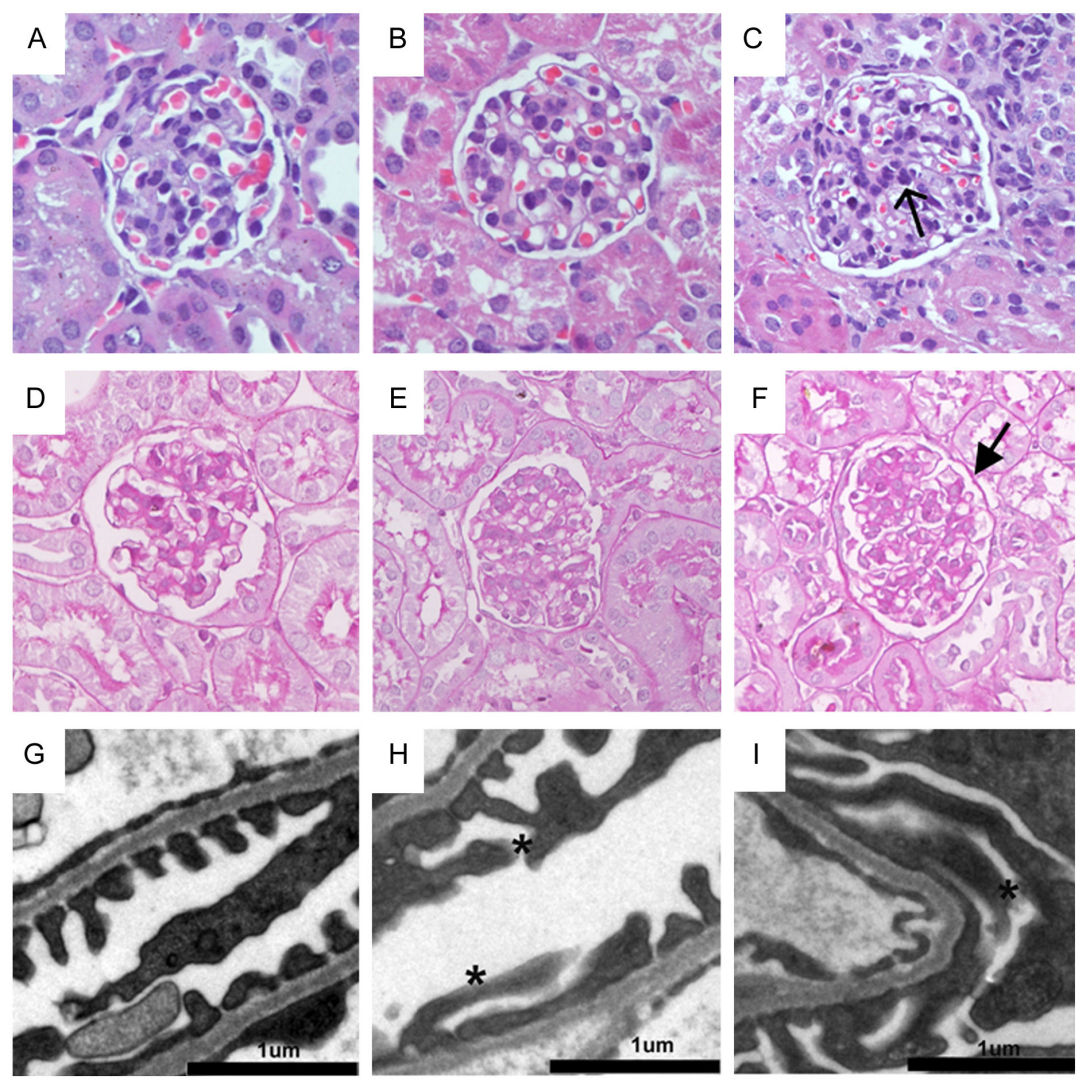
AA causes podocyte foot-process effacement in mice. (A-F) Representative micrographs show the morphology in mice at different time points after AA administration. Kidney sections from sham (A and D), day 3 after AA (B and E) and day 7 after AA (C and F) were subjected to hematoxylin-eosin (HE) (A-C) and periodic acid-schiff (PAS) (D-F) (magnification: ×400). (G-I) Representative electron micrographs show podocytes foot process structures. Scale bar, 1μm. Arrows indicate infiltrated cells. Stars show podocyte foot-process effacement.

Figure 2A to 2F show the representative micrographs of kidney sections from sham and mice receiving AA at day 3 and day 7 after administration. Kidney histology remained almost normal when compared with the sham mice. Because albuminuria always results from the damage of glomerular filtration barrier, we next examined the ultrastructural alternations of the filtration barrier. Glomerular filtration apparatus consist of the fenestrated endothelial cytoplasm, glomerular filtration barrier (GBM) and the foot processes of podocytes [18,19]. As shown in Figure 2G-2I, kidney endothelial cells and GBM were essentially normal as compared with sham. However, a marked ultrastructural alternation characterized by foot-process effacement was observed in mice received AA as early as day 3 after administration (Figure 2H and 2I). The adhesion of effaced foot-process to GBM led to damage and disappearance of the slit diaphragm, which is the major component of filtration barriers. The morphological changes of effacement of foot-process and resulted damage of slit diaphragm might explain the manifestation of albuminuria in mice received AA.

**Figure 3:**
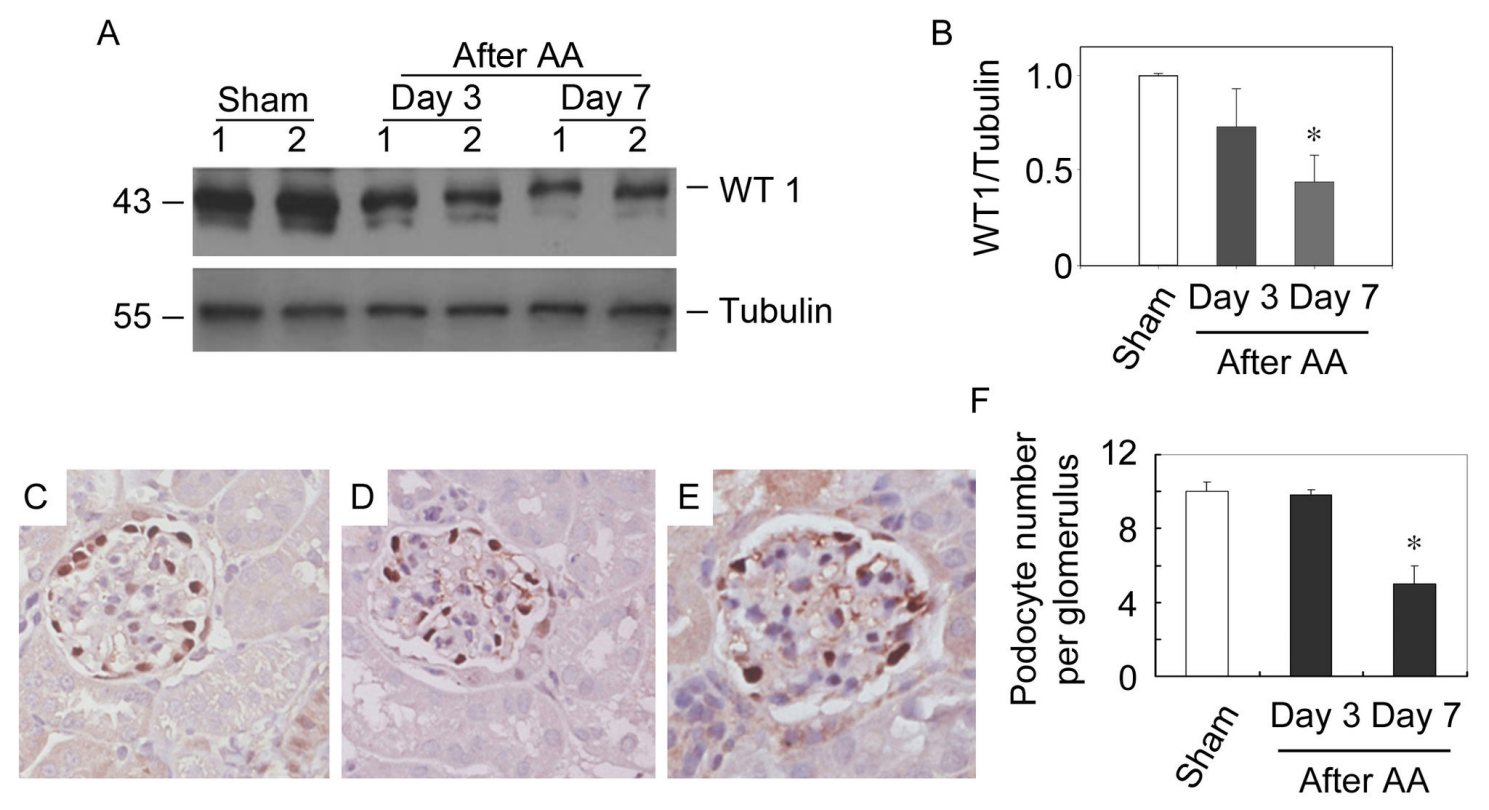
AA causes podocyte depletion in mice. (A) Western blot analysis of WT1 expression levels in mice received AA. Whole kidney lysates were immunobloted with antibodies against WT1 and Tubulin, respectively. Samples from two individual animals per group were used. (B) Graphic presentation of relative WT1 abundance normalized to Tubulin. * *P*<0.05 versus sham (*n* = 3). (C-E) Representative micrographs show the podocyte abundance at sham (C), day 3 after AA (D) and day 7 after AA (E). Kidney sections were stained with antibodies against WT1. (magnification: ×400) (F) Quantitative determination of the glomerular podocyte numbers at different time points after AA administration. * P < 0.05 versus sham (n = 4).

 We next examined whether podocyte foot-process effacement would ultimately lead to detachment of podocyte. The podocyte abundance was examined by quantification of WT1, a zinc finger protein that expresses restrictively by podocyte in mature kidney [20,21], at different time points after AA administration. Western blot analysis revealed that expression levels of WT1 in kidney were decreased time-dependently after AA. At day 7 after AA, more than 50% of WT1 protein abundance was lost (Figure 3A and 3B). This finding was further supported by quantitative determination of WT1-positive cell numbers per glomeruli in mice receiving AA at different time points (Figure 3C-3F). The number of podocytes per glomeruli was decreased by about 50% as compared with the sham. Together, podocyte foot-process effacement and detachment, which ultimately resulted in damaged glomerular filtration barrier, are the major causes of albuminuria that takes place in mice receiving AA.

### AA impairs the filtration barrier function of cultured podocytes

**Figure 4:**
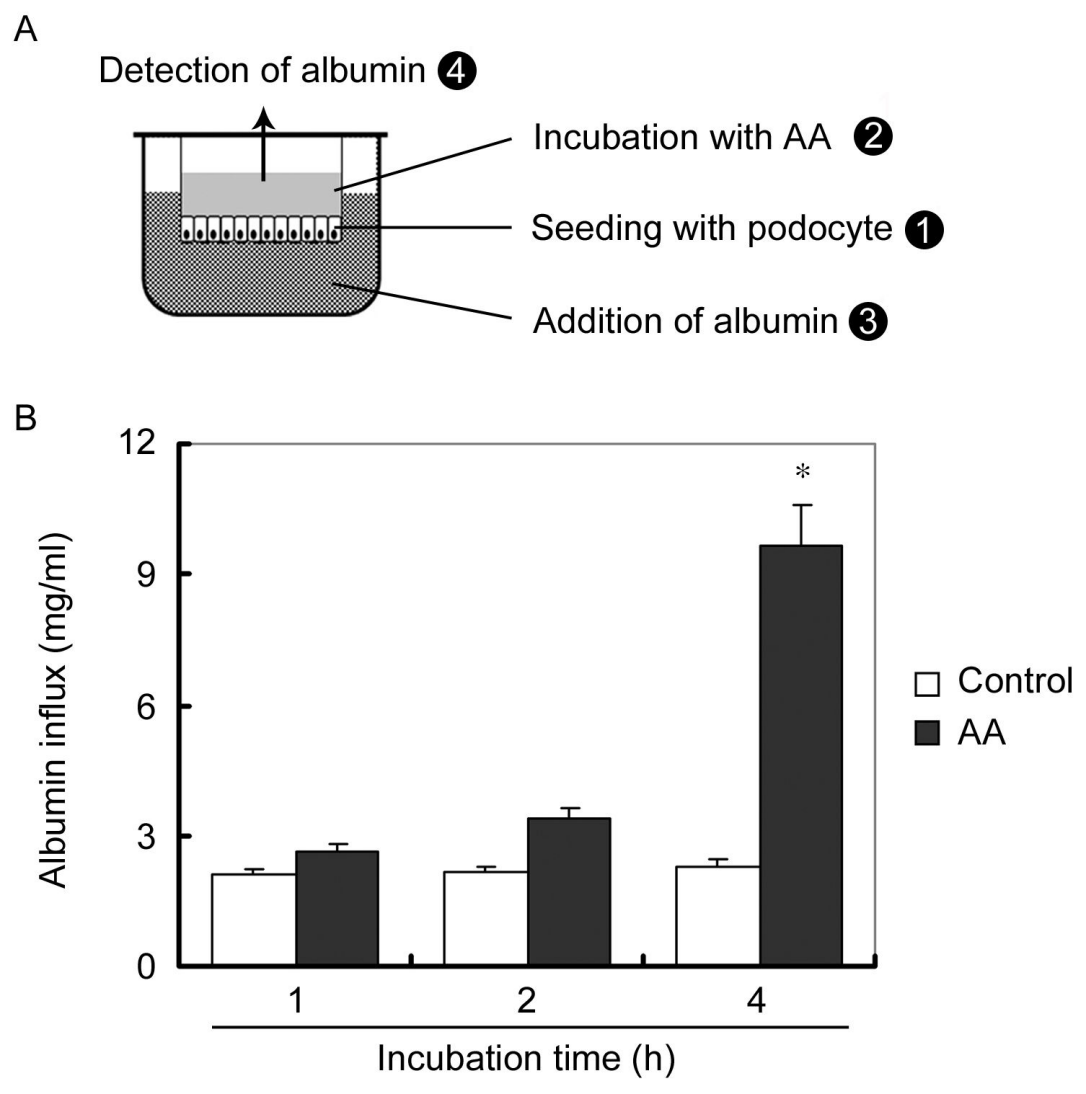
AA impairs the filtration barrier function of cultured podocytes. (A) Schematic depiction of the paracellular permeability influx assay. Podocyte monolayer on collagen-coated transwell filters was incubated without or with AA for 48 hours, and albumin permeability across podocyte monolayer was determined. (B) Graphic presentation of the albumin influx across podocyte monolayer. Duration of albumin incubation is shown on x-axis. Data are presented as mean ± SEM. * P < 0.05 versus control (n = 3).

 We next confirmed the impairment of AA on glomerular filtration barrier of podocytes using an in vitro paracellular permeability influx assay system. As illustrated in Figure 4A, podocytes were seeded on the upper chamber of the transwell. After the differentiated podocytes monolayer became confluent, 10µmol/L of AA was added into the upper chamber to induce podocyte injury. We hypothesized that the albumin added in the lower chamber would leak to the upper chamber if the slit diaphragm formed between foot-process of confluent podocytes were impaired. As we expected, compared with control, AA treatment resulted in a greater albumin influx across the podocyte monolayer (Figure 4B). These results indicate that filtration barrier function of cultured podocytes is impaired by AA, which is in accordance with the albuminuria in mice receiving AA.

### AA causes mitochondrial DNA damage of cultured podocytes

**Figure 5:**
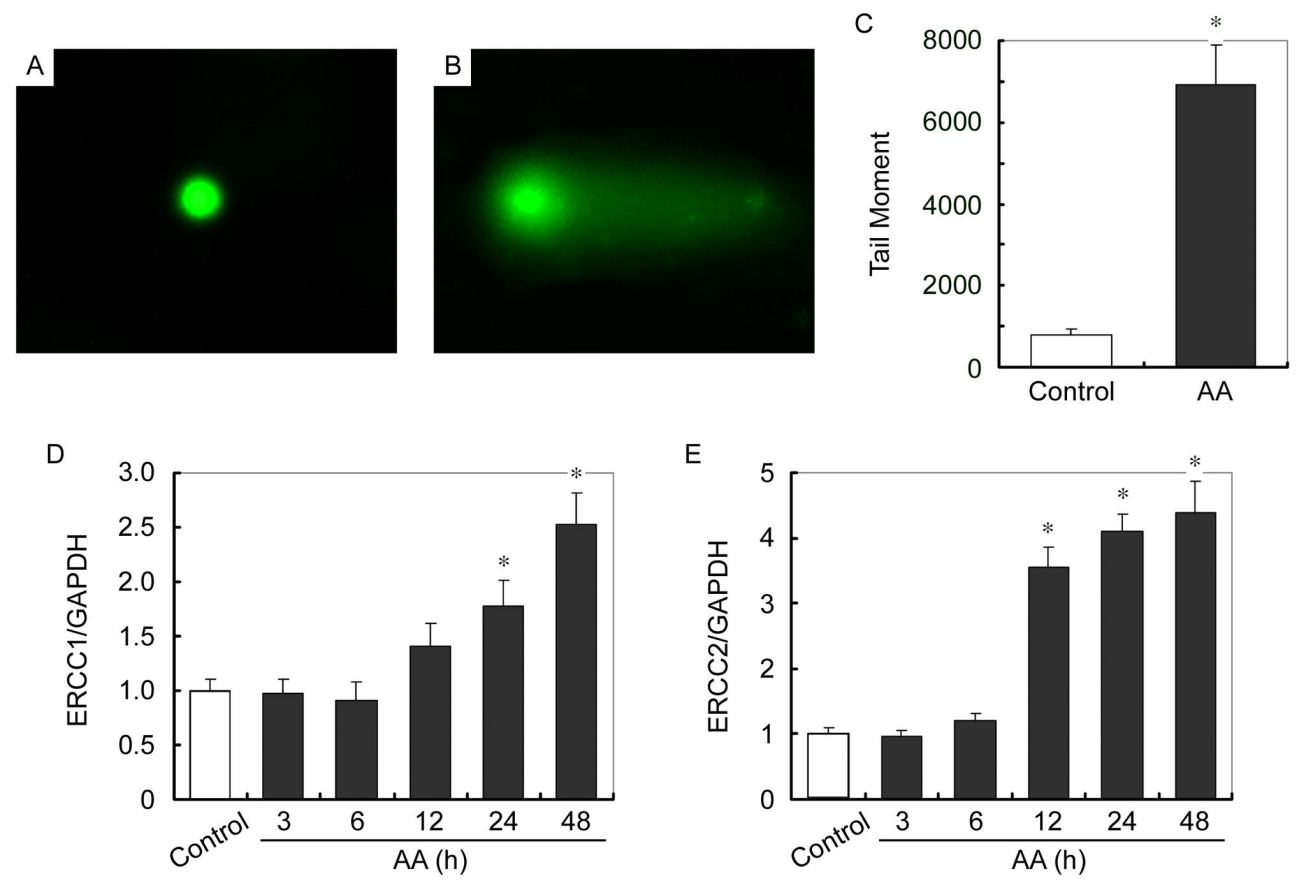
AA causes mtDNA damage of cultured podocytes. (A and B) Representative micrographs show the comet assay of mtDNA isolated from control (A) and 12h of AA-treated (B) podocytes. (C) Graphic presentation of Tail moment in control and AA treated podocytes. Tail moment was measured using tail DNA percent x tail moment length. Tail DNA percent was calculated by 100 x tail DNA immunofluorescent intensity / total DNA immunofluorescent intensity. * P < 0.05 versus control (n = 3). (D and E) Q-PCR analysis of ERCC1 (D) and ERCC2 (E) expression levels normalized to GAPDH in podocytes at different time points of AA treatment. * *P* < 0.05 versus control (*n* = 3).

Mitochondrial is the only organism other than nuclear that has inheritable DNA in eukaryotic cells, which is called mitochondrial DNA (mtDNA). It was reported that metabolized AA induced cell damage and carcinogenesis by forming DNA adducts in AAN patients [2,17,22,23]. We therefore tested whether mtDNA of podocytes was also the victim of AA injury. Mitochondria were isolated from AA-treated podocytes and mtDNA damage was evaluated using single cell gel electrophoresis (also called comet assay). As shown in Figure 5A and 5B, mtDNA of the AA-treated podocytes migrated further than that of control cells and produce a “comet tail” shape. DNA damage was quantified by measuring the displacement between the genetic material of the nucleus (comet “head”) and the resulting “tail”. Tail DNA percent was calculated by 100 x tail DNA immunofluorescent intensity / total DNA immunofluorescent intensity. Tail moment was measured using tail DNA percent x tail moment length (Figure 5C). 

We next examined whether AA impaired DNA repair by evaluating the levels of predominant DNA repair proteins, including excision repair cross-complement rodent repair deficiency, complementation group 1 and 2 (ERCC1 and ERCC2), methylguanine methyl transferase (Mgmt) and Poly [ADP-ribose] polymerase 1 (PARP1). As shown in Figure 5D to 5E, mRNA expression levels of ERCC1 and ERCC2 are markedly increased after AA treatment in a time-dependent manner. Expressions of Mgmt and PARP1 mRNAs were also increased, however not statistically significant (data not shown). Taken together, AA directly impairs mtDNA of podocytes without affecting DNA repair.

### AA reduces mitochondria biogenesis in cultured podocytes

**Figure 6:**
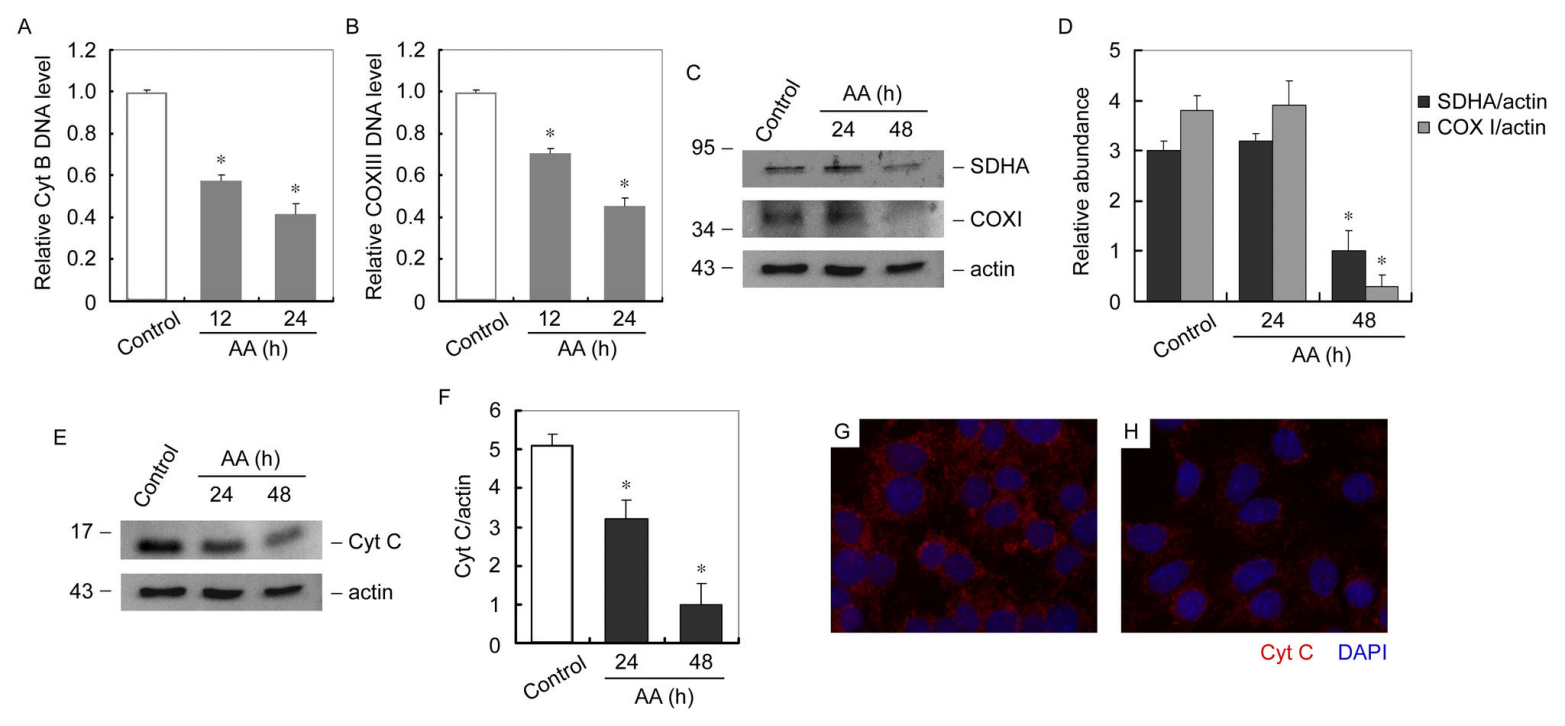
AA reduces mitochondria biogenesis in cultured podocytes. (A and B) Q-PCR analysis of relative Cyt B (A) and COX III (B) DNA expression levels in podocytes at different time points after AA treatment. * *P* < 0.05 versus control (*n* = 3). (C) Western blot analysis of mitobiogenesis levels in podocytes at different time points of AA treatment. Whole cell lysates was immunobloted with antibody against mitobiogenesis western blot cocktail (SDHA, COX I and actin). (D) Graphic presentation of relative SDHA and COX I abundance normalized to actin. * *P*<0.05 versus control (*n* = 3). (E) Western blot analysis of Cyt C levels in podocytes at different time points of AA treatment. Whole cell lysates was immunobloted with antibody against Cyt C. (F) Graphic presentation of relative Cyt C abundance normalized to actin. * *P*<0.05 versus control (*n* = 3). (G and H) Representative micrographs show immunofluorescent staining of Cyt C (red) in control and 24h of AA treated podocytes. Nuclei were stained with DAPI (blue) (magnification: ×400).

 Since AA damaged mtDNA, we hypothesize that mitochondrial biogenesis could be impaired in AA treated podocytes. Mitochondrial biogenesis was evaluated by quantification of mtDNA copy number, mitochondrial respiratory protein subunits and mitochondrial number. MtDNA copy number was evaluated by measuring DNA levels of cytochrome B (Cyt B) and cytochrome oxidase III (COX III) [24,25]. As shown in Figure 6A and 6B, compared with control podocytes, mtDNA copy number was markedly decreased time-dependently after AA incubation. Mitochondrial respiratory protein subunits were encoded either by mtDNA or nuclear DNA (nDNA). Expression of mitochondrial respiratory protein subunits was examined by immunoblot analysis of nDNA-encoded SDHA and mtDNA-encoded COXI. As shown in Figure 6C, expressions of respiratory proteins were markedly reduced in cultured podocytes after 48h of AA treatment. Cytochrome C (Cyt C) is a component of the electron transport chain in mitochondria, whose expression level also indicates the quantity of mitochondria. We therefore examined the Cyt C expression in AA treated podocytes. As shown in Figure 6E, expression levels of Cyt C were decreased time-dependently in AA treated podocytes. Immunofluorescent staining revealed that Cyt C was largely reduced after AA treatment (Figure 6G and 6H). 

### AA impairs mitochondrial functions in cultured podocytes

**Figure 7:**
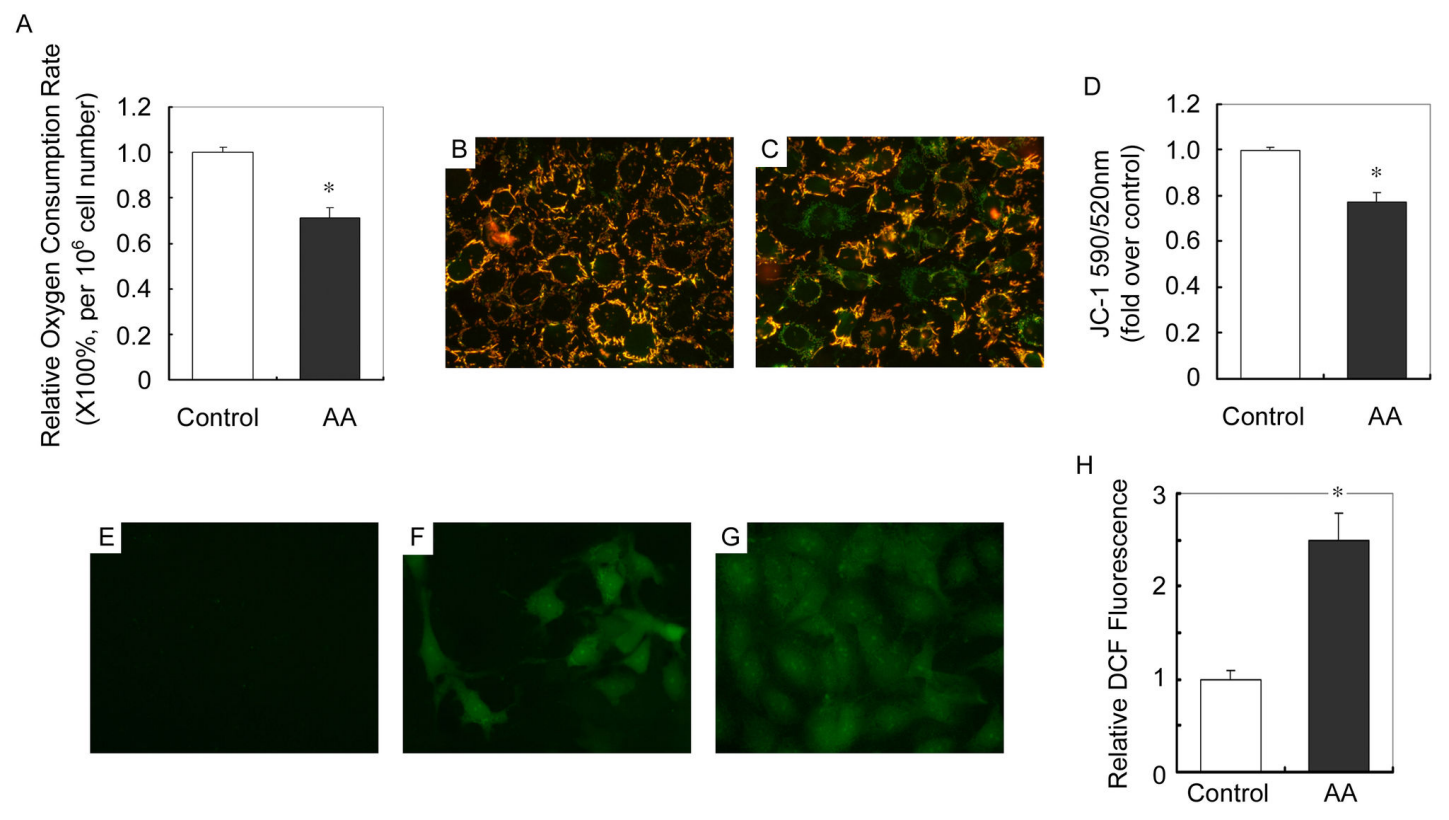
AA impairs mitochondrial functions in cultured podocytes. (A) Graphic presentation of the relative oxygen consumption rate in control and 12h of AA treated podocytes. * *P*<0.05 versus control. (n = 3) (B and C) Representative pictures show JC-1 staining in control (B) and 12h of AA (C) treated podocytes (magnification: ×400). (D) Graphic presentation of the ratio of J-aggregate/monomer (590/520 nm) in control and 12h of AA treated podocytes. Values are expressed as the fold over control cells. * *P*<0.05 versus control (n = 3). (E-G) Representative pictures show ROS production in control (E) and 12h of AA (F) treated podocytes as measured by DCF fluorescence (magnification: ×400). (G) Positive control staining of DCF fluorescence. (H) Graphic presentation of relative DCF fluorescence in control and 12h of AA treated podocytes. * *P*<0.05 versus control (n = 3).

We next determined whether damaged mtDNA and further impaired mitochondrial biogenesis would result in mitochondrial dysfunction. Mitochondrial respiratory function (aerobic respiration) was evaluated by oxygen consumption rate as calculated by examining the degression rate of the concentration of dissolved oxygen in cell culture media. Figure 7A shows that after 12h of AA treatment, oxygen consumption rate was markedly decreased as compared with control.

Maintenance of mitochondrial membrane potential reflects mitochondrial function. In this study, mitochondrial membrane potential in cultured podocytes was detected by staining of JC-1, whose transformation from J-aggregates to monomer, detected by fluorescence changes from red to green, reflected the decrease of membrane potential. As shown in Figure 7B and 7C, after 12h of AA treatment, mitochondrial potential was markedly decreased as compared with control.

Moreover, dysfunction of mitochondria always results in an increase of reactive oxygen species (ROS) production. AA-induced ROS in cultured podocytes was determined by dihydroethidine (DCF) fluorescence staining. AA treatment resulted in a more than twofold increase in DCF fluorescence in podocytes than in control cells (Figure 7E, 7F and 7H).

### AA causes mtDNA damage in mice kidneys

**Figure 8:**
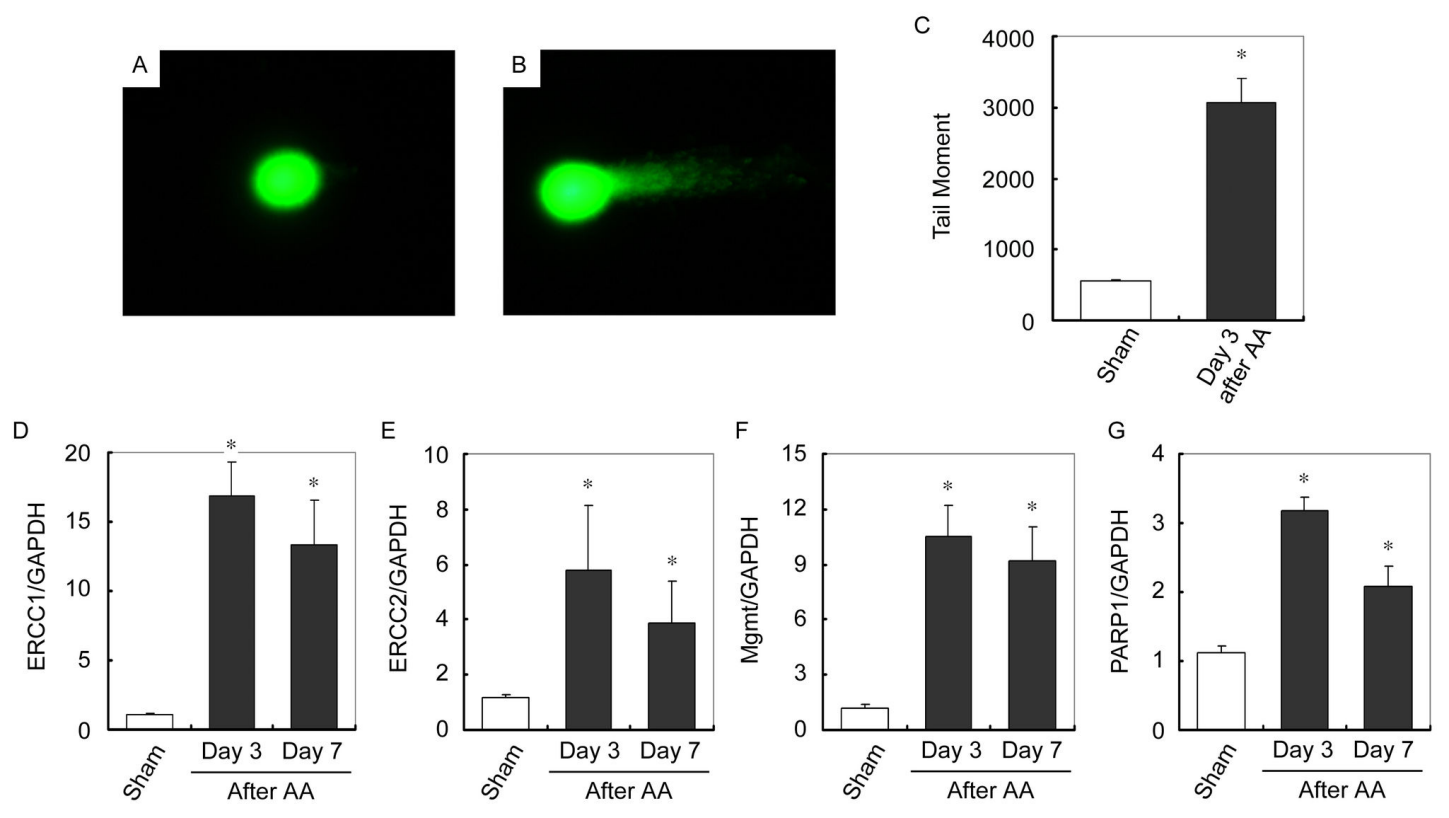
AA causes mtDNA damage in mice kidneys. (A and B) Representative micrographs show the comet assay of mtDNA isolated from mice kidneys of sham (A) and day 3 after AA (B). (C) Graphic presentation of Tail moment of mtDNA in kidneys of sham and day 3 after AA. * P < 0.05 versus sham (n = 7). (D-G) Q-PCR analysis of ERCC1 (D), ERCC2 (E), Mgmt (F) and PARP (G) expression levels normalized to GAPDH in sham and different time points after AA administration. * *P* < 0.05 versus sham (*n* = 4).

MtDNA damage was thereafter verified in kidney of mice received AA. Mitochondria were isolated from kidney and mtDNA damage was evaluated using comet assay. As shown in Figure 8A and 8B, mtDNA isolated from kidney of mice received AA migrated further than that of sham and produce a “comet tail” shape. DNA damage was quantified by tail moment, which revealed a markedly increased damage of mtDNA in kidneys of mice received AA compared with that in kidneys of sham (Figure 8C). Similarly as in cultured podocytes, transcription levels of DNA repair proteins, including ERCC1. ERCC2, Mgmt and PARP1 were examined. Figure 8D to 8G show that mRNA expression levels of ERCC1. ERCC2, Mgmt and PARP1 are all markedly increased in kidneys in mice received AA. Taken together, AA directly causes mtDNA damage without affecting DNA repair in mice kidney.

### AA reduces mitochondria biogenesis in mice kidneys

**Figure 9:**
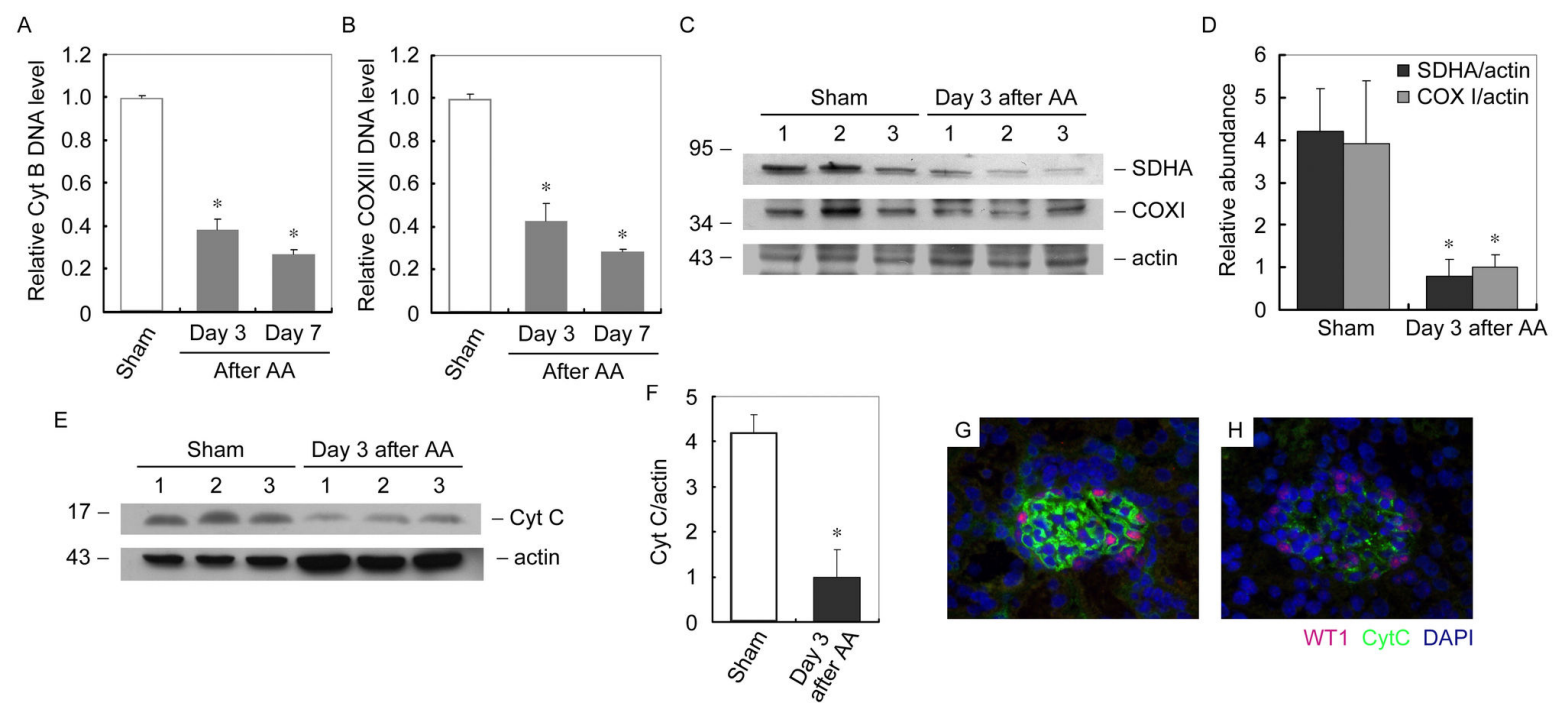
AA reduces mitochondria biogenesis in mice kidneys (A and B) Q-PCR analysis of relative Cyt B (A) and COX III (B) DNA expression levels in kidney at different time points after AA administration. * P < 0.05 versus sham (n = 4). (C) Western blot analysis of mitobiogenesis levels in kidney at different time points after AA administration. Whole kidney lysates was immunobloted with antibody against mitobiogenesis western blot cocktail (SDHA, COX I and actin). (D) Graphic presentation of relative SDHA and COX I abundance normalized to actin. * P<0.05 versus sham (n = 4). (E) Western blot analysis of Cyt C levels in kidney at different time points after AA administration. Whole kidney lysates were immunobloted with antibody against Cyt C and actin. (F) Graphic presentation of relative Cyt C abundance normalized to actin. * P<0.05 versus control (n = 4). (G and H) Representative micrographs show immunofluorescent staining of Cyt C (green) in kidney at day 3 after AA administration. (G) Sham. (H) Day 3 after AA. Podocytes were identified as WT1 (red)-positive cells. Nuclei were stained with DAPI (blue) (magnification: ×400).

 We further examined mitochondrial biogenesis in mice kidney after receiving AA. As shown in Figure 9A and 9B, compared with sham, mtDNA copy number was markedly decreased time-dependently in kidney of mice received AA. Expressions of mitochondrial respiratory protein subunits, SDHA and COXI, were markedly reduced at day 3 in kidney of mice received AA (Figure 9C). Expression level of Cyt C were decreased at day 3 in kidney of mice received AA (Figure 9E).Immunofluorescent staining also confirmed that Cyt C was largely reduced in glomeruli of mice received AA (Figure 9G and 9H). 

## Discussion

AAN was previously identified as a progressive renal disease characterized by tubulointerstitial fibrosis and urothelial cancer caused by a nature herb namely aristolochic acid [2]. However, the presence of albuminuria in some patients with AAN and animal received AA [12,13,15,16] suggests that glomerular filtration barrier might also be the victim after in-taking of AA. In this study, mice administered with high dose (6 mg/kg body weight) of AA rapidly developed albuminuria (Figure 1). Moreover, the severity of albuminuria was specifically dependent on the dosage that administered. In the kidney, ingredients of proteinuria reflect the damaged segments and albuminuria always suggests the pathological changes of glomerular filtration barrier. The glomerular filtration barrier consists of fenestrated endothelial cytoplasm, glomerular filtration barrier (GBM) and the foot processes of podocytes. Histological staining showed almost normal glomeruli after AA administration (Figure 2A-2F). However, electronic microscopy revealed that podocyte foot-process effacement other than GBM impairment might probably contribute to the leakage of albuminuria (Figure 2G-2i). Since podocyte foot-process is a pivotal component, disruption of its integrity represents the key development of albuminuria in a variety glomerular disease [18,26]. It was found in this study that AA could induce glomeruli damage besides tubulointerstitial fibrosis, and the impairment of podocyte foot-process mainly explained the incidence of albuminuria in mice received AA.

 In the transwell cell culture system, a model of glomeruli filtration barrier consisted of podocytes and GBM was applied to confirm the effect of AA on podocytes. After the differentiated podocyte monolayer became confluent, the slit diaphragm between foot processes could theoretically restrict the leakage of albumin [27,28]. As we expected, podocyte foot-process was impaired after AA treatment as demonstrated by markedly increased albumin leakage from the lower chamber (plasma) to the upper chamber (urine) (Figure 4). This in vitro simplified paracellular permeability influx assay system helped us confirmed that AA could damage podocyte foot-process and thereafter induce albuminuria.

 AA is widely presented in many nature plants [6,7,9]. Once intake, several enzymes have demonstrated to metabolize AA into a cyclic N-acylnitrenium ion with delocalized positive charge able to covalently bind to exocyclic amion groups of purine bases and form DNA adducts [2]. DNA adducts have already been retrieved in AAN patients [17]. In eukaryocytes, the majority of DNA exists in the nuclei; however, mitochondrion is the other and the only organism that has inheritable DNA, which is called mitochondrial DNA (mtDNA). As the metabolic factory, mitochondrion is important for the wellbeing of eukaryocyte. As for mitochondrion, mtDNA is indispensable, since mtDNA encodes several mitochondrial respiratory proteins and controls their expression. We therefore determined whether mtDNA of podocyte was also the victim of AA injury. Comet assay of mitochondria isolated from cultured podocytes revealed that AA treatment indeed induced mtDNA damage (Figure 5A and 5B). Under an electrophoretic field, the different sides of DNA fragments and strand breaks are separated from intact DNA, yielding a classic “comet tail” shapes under the microscope (Figure 5C). DNA damage occurred due to environmental factors and metabolic processes at a rate of 1,000 to 1,000,000 molecular lesions per cell per day. We therefore examined whether the markedly increased mtDNA damage was caused by impaired DNA repair system. MRNA levels of several predominant DNA repair proteins, including ERCC1, ERCC2, Mgmt and PARP1 was examined. Among them, ERCC1 and ERCC2 mRNA levels were markedly up-regulated after AA treatment (Figure 5D and 5E). Taken together, AA directly induced mtDNA damage in cultured podocytes without affecting DNA repair. Furthermore, AA-induced mtDNA damage was also confirmed in mitochondria from kidneys of mice received AA (Figure 8A-8C). Similarly as in cultured podocytes, up-regulation of mRNA levels of ERCC1, ERCC2, Mgmt and PARP1 after AA was unaffected (Figure 8D-8G). 

 Not surprisingly, after the damage of mtDNA, mtDNA copy number, mitochondrial protein and mitochondrial number were all markedly decreased in podocytes treated with AA (Figure 6) and kidneys of mice received AA (Figure 9). Moreover, AA impaired mitochondrial functions, including aerobic respiration, mitochondrial membrane potential and ROS production of podocyte (Figure 7). These results depicted a process occur in podocytes after the administration of AA. After been absorbed, metabolized AA damages mtDNA probably by forming DNA adducts. Although the DNA repair system is stimulated, the AA-induced mtDNA damage seemed to be irreversible since the mtDNA copy number decreased with the time being. Moreover, decrease of mtDNA-encoded COX I and nuclei DNA-encoded SDHA suggested that mtDNA and nuclei DNA were both victims of AA. In addition, mitochondrial functions that related to cell fate were markedly impaired. All together, the podocytes detachment in kidneys after AA administration was reasonable (Figure 3). The nuclei DNA damage was also observed in kidneys of mice received AA and podocytes treated with AA (data not shown), and the contributions of nuclei DNA damage to podocytes injury and albuminuria could hardly be eliminated. However, the abnormality of mtDNA biogenesis (Figures 6 and 9) and mitochondrial functions (Figure 7) were probably attributed to mtDNA damage.

 In this study, it was demonstrated that AA-induced albuminuria is caused by podocyte damage other than previously acknowledged tubulointerstitial fibrosis in AAN. Moreover, DNA outside the nuclei is also the target of AA. Similarly as nuclei DNA, damage of mtDNA by AA, which thereafter causes mitochondria dysfunction, contributes to the pathogenesis of podocyte foot-process effacement and detachment. This study highlights the podocytes and mtDNA in the pathogenesis of AA-induced kidney injury.

## Material and Methods

### Ethics statement

All of the following details of the study were approval by institutional review board of Nanjing Medical University.

### Animal model

Experiments were performed on 6 to 8 weeks old male CD-1 mice that weighed ~ 20g. Mice were housed in the animal facilities of National Model Animal Center (Nanjing, Jiangsu Province, China) with free access to food and water. They were treated humanely in accordance with NMAC guidelines and approved protocols of the Institutional Animal Use and Care Committee at the Nanjing Medical University. Aristolochic acid (AA) I sodium salt (≥97%) was purchased from Sigma-Aldrich (A9451). AA was dissolved in saline to a concentration of 1mg/ml and daily administered intraperitoneally at a dose of 6mg/kg bodyweight for 3 days. Same volume of saline with adjusted pH value was administered in sham mice. Mice were randomly allocated into three groups: sham (n=7), day 3 after AA (n=7) and day 7 after AA (n=7). Mice were killed at day 3 and day 7 after AA as indicated (Figure 1A). 

### Cell culture and treatment

 Mouse conditionally immortalized podocyte cell line was cultured in RPMI 1640 medium supplemented with 10% fetal bovine serum (FBS), (Invitrogen), 100U/ml penicillin, 100U/ml streptomycin and 50U/ml mouse recombinant γ-interferon at 33°C. To induce differentiation, podocytes were cultured at 37°C in the absence of γ-interferon for 10-14 days before harvested for experiment. Podocytes grown under differentiating condition were seeded at 80% confluence in complete medium containing 10% FBS. After 24 hours, the cells were changed to serum-free medium and incubated for additional 16 hours. For AA treatment, cells were incubated with 10µmol/L of AA (Sigma-Aldrich, A9451) for various time periods.

### Urine albumin and creatinine assay

Urine albumin was measured by using a mouse albumin ELISA quantization kit according to manufacture’s protocol (Bethyl Laboratories). Urine creatinine was determined by using a commercial kit (Bioassay Systems, DICT-500). Urine samples at different time point were separated on 10% SDS-PAGE after correction to creatinine and visualized after been stained with coomassie brilliant blue (Beyotime). BSA (1μg) was loaded as positive control.

### Histology staining

For light microscopy, kidney sections were prepared at 3μm thickness by a routine procedure. Sections were stained with hematoxylin-eosin (HE) and periodic acid-schiff (PAS) by standard protocol. The histologic sections were examined by a renal pathologist. For electron microscopy, kidney sections were fixed in 2.5% glutaraldehyde in phosphate buffer (pH 7.4) and postfixed with 1% osmium tetroxide. Fixed tissue blocks were dehydrated and embedded in Epon 812. Ultrathin sections were stained with uranyl acetate and lead citrate and then examined under a transmission electron microscope.

### Albumin Influx Assay

To evaluate the filtration barrier function of podocyte monolayer, differentiated podocytes (5×10^3^) were seeded on the collagen-coated transwell filters (3μm pore; Corning) at the top chamber. Podocytes were then changed to serum-free medium overnight. After been treated with 10µM of AA for 48 hours, podocytes were washed twice with PBS supplemented with 1mM MgCl_2_ and 1mM CaCl_2_ to preserve the cadherin-based junctions. The top chamber was then refilled with serum-free medium and the bottom chamber was filled with medium supplemented with 40mg/ml of bovine serum albumin. The podocytes were incubated at 37°C and the aliquot of medium from top chamber was collected at different time periods as indicated for albumin concentration measurement by using a Bio-Rad protein assay kit (Bio-Rad Laboratories , Hercules, CA).

### Western immunoblot analysis

Kidney tissue was homogenized by a polytron homogenizer (Brinkmann Instruments, Westbury, NY) in RIPA lysis buffer on ice. Cells were lysed with sodium dodecyl sulfate (SDS) sample buffer. The primary antibodies used were as follows: anti-WT1 antibody (sc-7385, Santa cruz), mitobiogenesis western blot cocktail (SDHA, COXI and actin, ab123545; Abcam), anti-cytochrome C (Cyt C) (sc-8385; Santa Cruz), anti-Tubulin (T6199; Sigma-Aldrich) and anti-actin (sc-1616; Santa Cruz). Western blot were performed at least three times independently. Quantification was performed by measurement of the intensity of the signals with the aid of National Institutes of Health Image software package.

### Immunohistochemical staining for WT1

Kidney sections (3μm) were deparaffinized and rehydrated by xylene, a graded alcohol series and double deionized water. Briefly, after blocked with blocking buffer for 30min at room temperature, sections were incubated with anti-WT1 mouse monoclonal antibody (sc-7385, Santa cruz), then with secondary antibody of HRP-conjugated rabbit anti-mouse IgG.

### Immunofluorescent staining

Indirect immunofluorescent staining was performed according an established procedure [18]. Briefly, cells cultured on cover slips were washed twice with cold PBS and fixed with cold methanol/acetone (1:1) for 10 minutes at –20°C. Following three extensive washings with PBS, the cells were blocked with 0.1%TritonX-100 and 2% normal donkey serum in PBS buffer for 40 minutes at room temperature and then incubated with the specific primary antibodies described above, followed by staining with FITC or TRITC conjugated secondary antibody. Cells were double stained with DAPI to visualize the nuclei. Slides were viewed with a Nikon Eclipse 80i Epi-fluorescence microscope equipped with a digital camera (DS-Ri1, Nikon). In each experimental setting, immunofluorescent images were captured with identical exposure settings.

### Mitochondria isolation

Mitochondria are isolated from mice kidney or cultured podocytes using differential centrifugation as described previously [19]. The key steps are as follows: rupturing of cells by mechanical and chemical means, differential centrifugation at low speed to remove debris and large cellular organelles and centrifugation at higher speed to isolate and collect mitochondria. The yield intact mitochondria were than applied for mtDNA damage analysis.

### DNA damage analysis (Comet assay)

DNA damage was measured by using a comet assay kit according to manufacture’s protocol (STA-350, OxiSelect). Briefly, isolated mitochondria are washed and resuspended in ice-cold PBS, and then mixed with molten agarose before application to the OxiSelect™ Comet Slide. These embedded mitochondria are then treated with a lysis buffer and alkaline solution, which relaxes and denatures the DNA. Finally, the samples are electrophoresed in a horizontal chamber to separate intact DNA from damaged fragments. Following electrophoresis, the samples are dried, stained with Vista Green DNA dye, and visualized by epi-fluorescence microscopy using a FITC filter. Immunofluorescent images were captured with identical exposure settings.

### Mitochondrial DNA copy number assay

Mitochondrial DNA (mtDNA) copy number analysis was performed by quantification of mitochondrial Cyt B and COX III levels, which are encoded by mtDNA. Tissue total DNA was isolated using DNeasy blood & tissue kit (69504, QIAGEN). Mitochondrial DNA was quantified by Q-PCR with specified primers to Cyt B and COX III acquired from QIAGEN.

### Quantitative polymerase chain reaction

Total RNA was prepared using a TRIzol RNA isolation system according to the instructions by the manufacturer (Invitrogen). The first strand of cDNA was synthesized using 1 μg of RNA in 20 μl of reaction buffer using MLV-RT (Promega, Madison, WI) and random primers at 42°C for 30 minutes. Quantitative polymerase chain reaction (Q-PCR) was performed using an Applied Biosystems 7300 Sequence Detection system. The C_T_ data were determined using default threshold settings and the mean C_T_ was determined from the duplicate PCRs. The ratios to sham group were calculated by using the equation 2^-ΔCT^, in which ΔC_T_ = C_T Sample_-C_T sham_. All the primers were acquired from QIAGEN.

### Oxygen consumption rate assay

 Cell Oxygen consumption rate was evaluated by measuring the degression rate of the concentrations of dissolved oxygen in cell culture media after different treatment as indicated.

### Mitochondrial membrane potential assay (JC-1 staining)

Mitochondrial membrane potential was monitored using JC-1, which is a membrane potential sensitive fluorescent dye. JC-1 staining was performed according to the instructions provided by the manufacture (C2005, Beyotime). After been treated differently, cells were stained, washed and observed by a Nikon Eclipse 80i Epi-fluorescence microscope equipped with a digital camera (DS-Ri1, Nikon). Podocytes were detected by fluorescence-assisted cell sorting using flow cytometer. The relative membrane potential was calculated using the ratio of J-aggregate/monomer (590/520 nm). Values are expressed as the fold over control cells.

### ROS production assay

ROS production of podocytes was measured by DCF fluorescence according to the instructions by the manufacturer (S0033, Beyotime). Control and treated cells were stained, washed and observed by a Nikon Eclipse 80i Epi-fluorescence microscope equipped with a digital camera (DS-Ri1, Nikon). Podocytes were also detected by fluorescence-assisted cell sorting using flow cytometer. The relative DCF fluorescence was calculated and expressed as the fold over control cells.

### Statistical analysis

Statistical analysis was performed using SigmaStat software (Jandel Scientific Software, San Rafael, CA). Comparisons between groups were made using one-way ANOVA, followed by the t test. P<0.05 was considered significant.
